# Supplementary figures and images for: The transcription factor TaWRKY58 coordinates growth and drought sensitivity in wheat by repressing TaLRR and TaBCS1
Source: PLoS Genet. 2026 Apr 24;22(4):e1012124. doi: 10.1371/journal.pgen.1012124 (PMC13132433; doi:10.1371/journal.pgen.1012124)

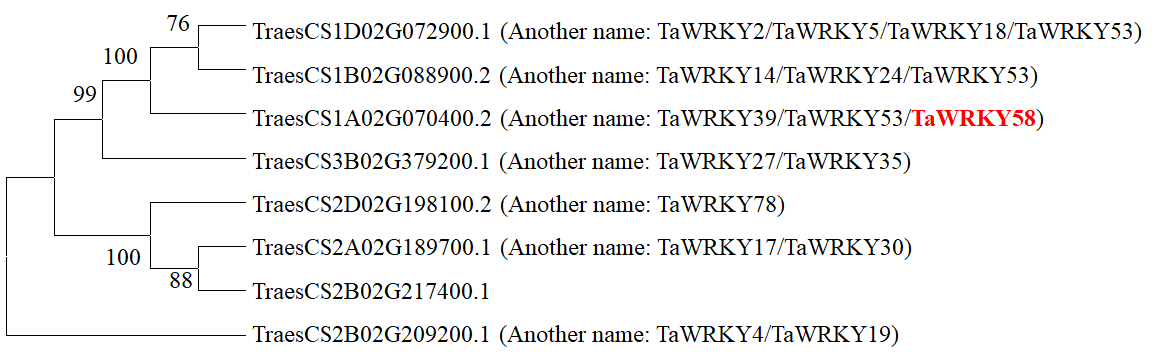

Supplement: S1 Fig — The reported protein sequences and classification basis are as described previously [3]. The unreported protein sequences were obtained through Blast search of the reported protein sequences in EnsemblPlants (http://plants.ensembl.org/index.html), and all were confirmed through domain prediction. Bootstrap test of phylogeny was conducted with 10,000 replicates, and branch length values were displayed at the nodes of each proposed class. (TIF) [file pgen.1012124.s002.tif]

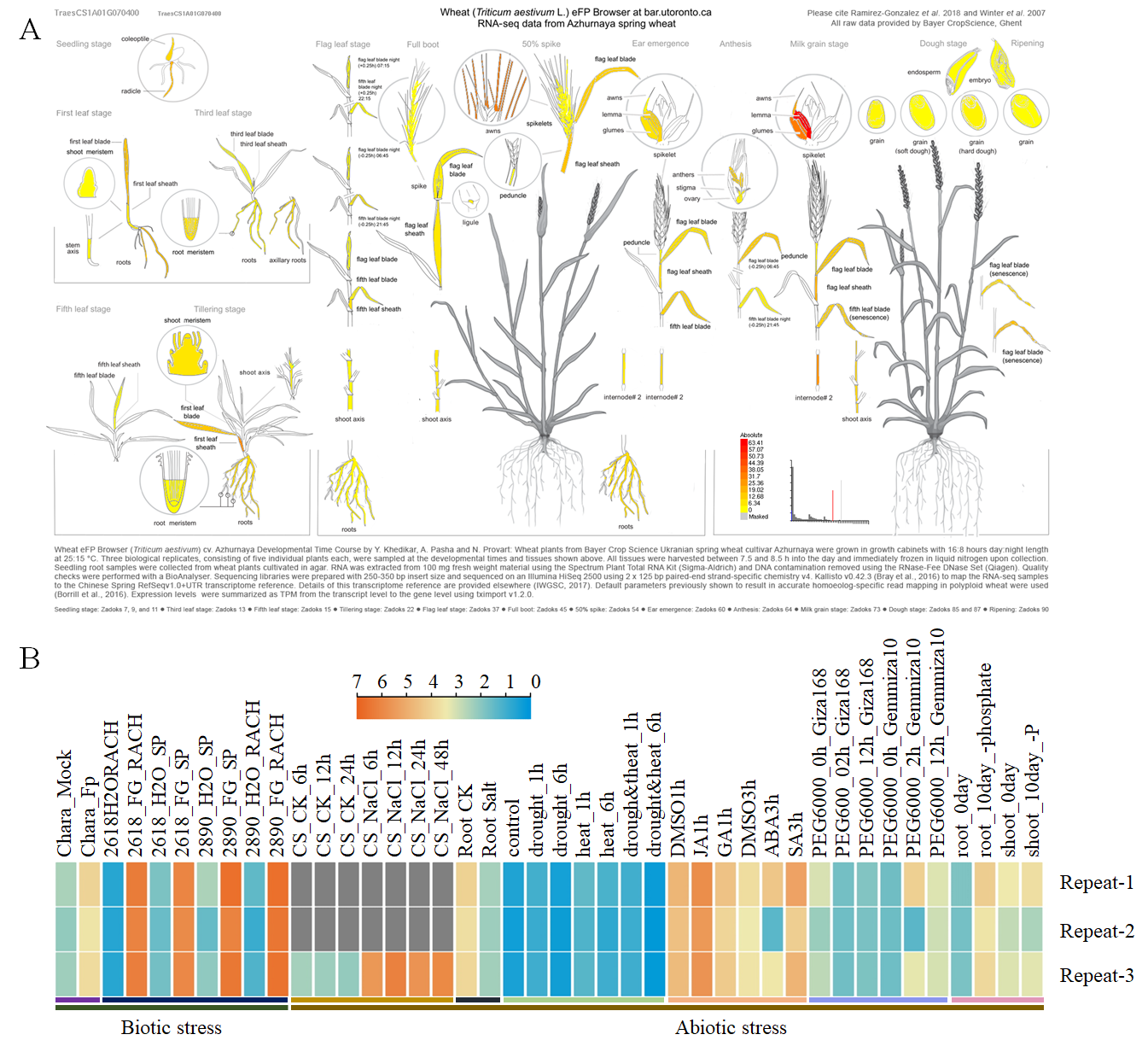

Supplement: S2 Fig — A. The transcription levels (log2FC) of TaWRKY58 during wheat development based on public database (The Bio-Analytic Resource for Plant Biology: https://bar.utoronto.ca/). B. The transcription levels (log2FC) of TaWRKY58 under Fusarium pseudograminearum (Fp) and Fusarium graminearum (Fg) infection, NaCl, PEG6000, Phosphate (Pi), jasmonic acid (JA), abscisic acid (ABA) and salicylic acid (SA) stress based on WheatOmics 1.0 database [36]. (TIF) [file pgen.1012124.s003.tif]

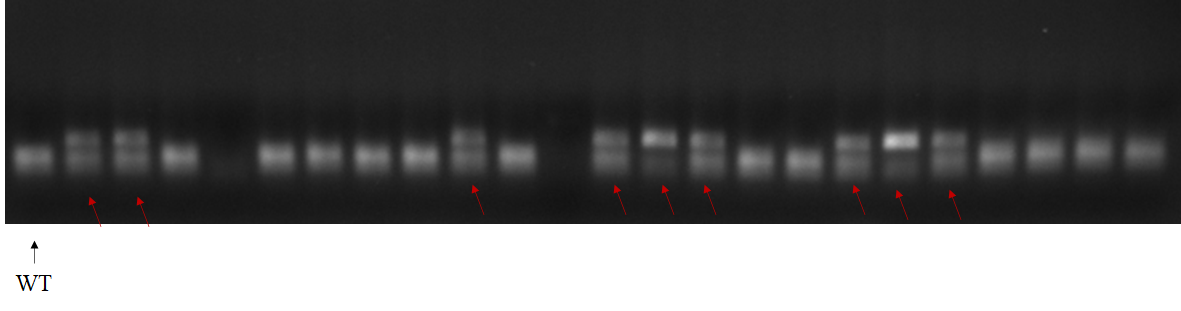

Supplement: S3 Fig — The red arrow represents Δtawrky58 mutant. The primers sequence listed in S1 Table. (TIF) [file pgen.1012124.s004.tif]

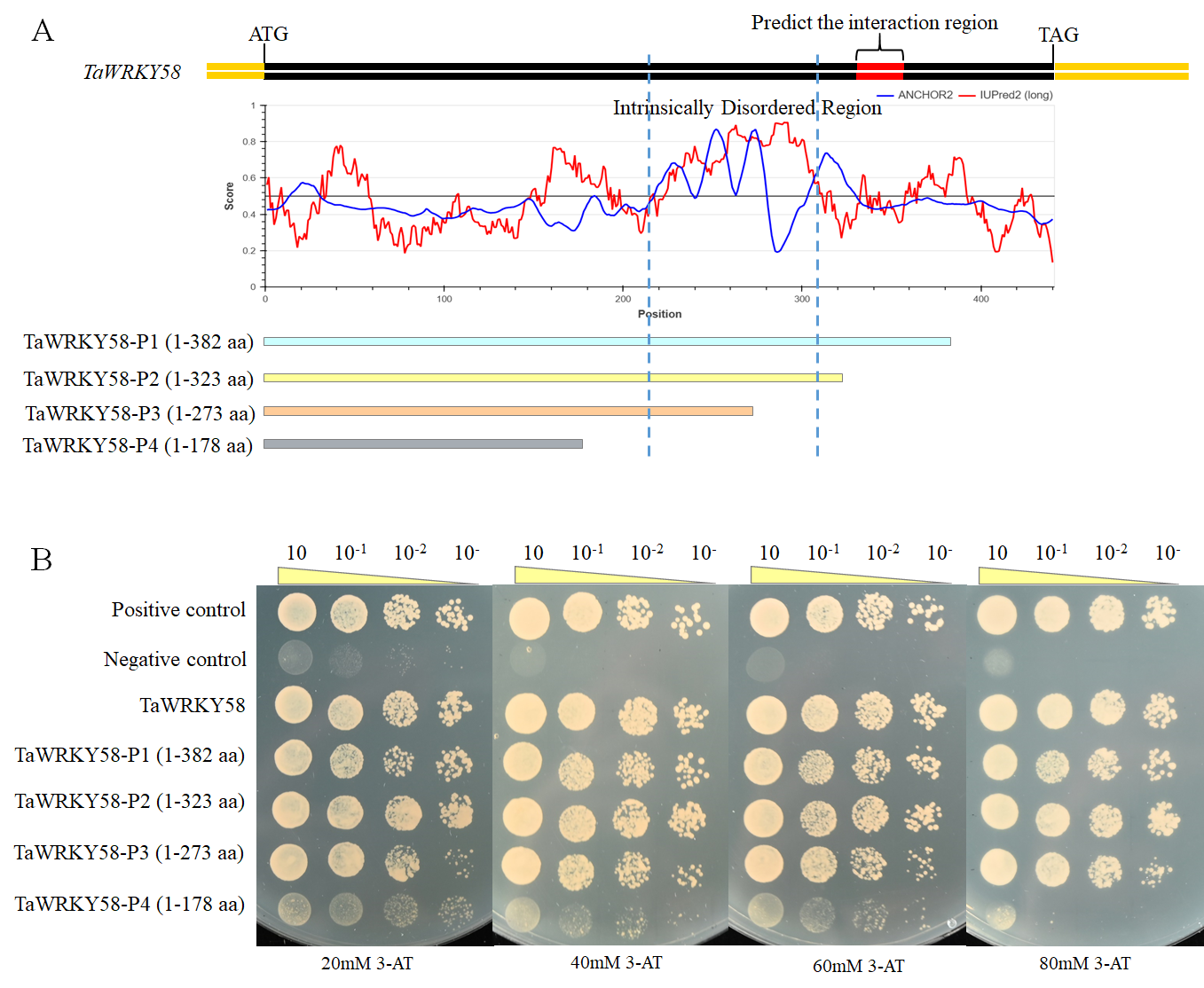

Supplement: S4 Fig — A. Prediction of the self-activation region of TaWRKY58 and schematic diagram of the positions of different fragments. B. Auto-activation assay of TaWRKY58 in yeast. (TIF) [file pgen.1012124.s005.tif]

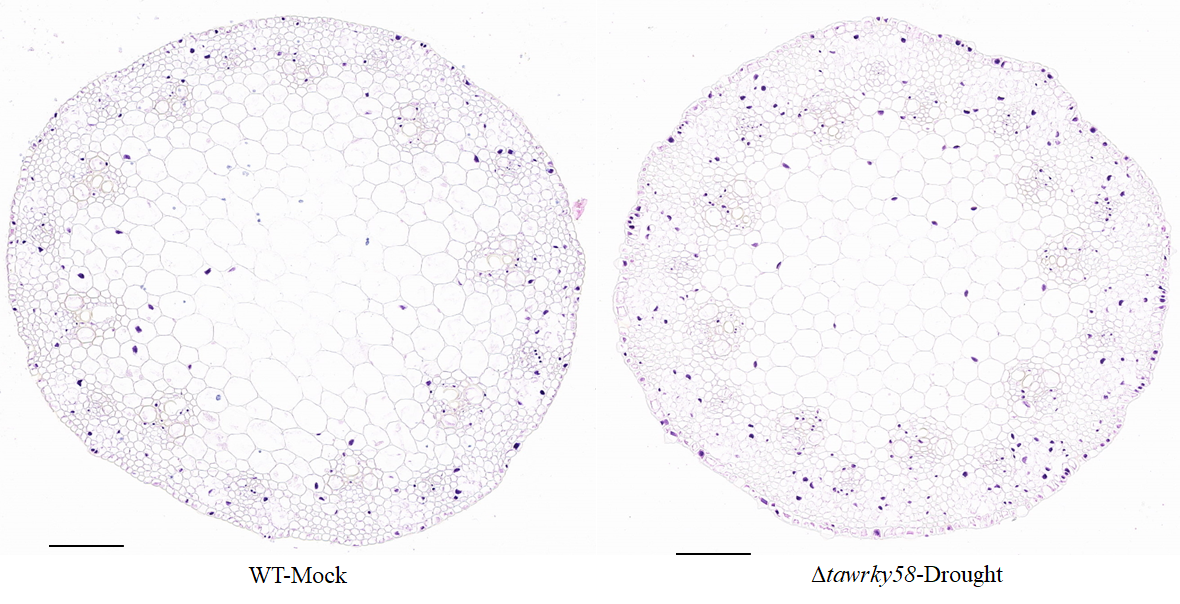

Supplement: S5 Fig — Scale bar = 100 μm. (TIF) [file pgen.1012124.s006.tif]

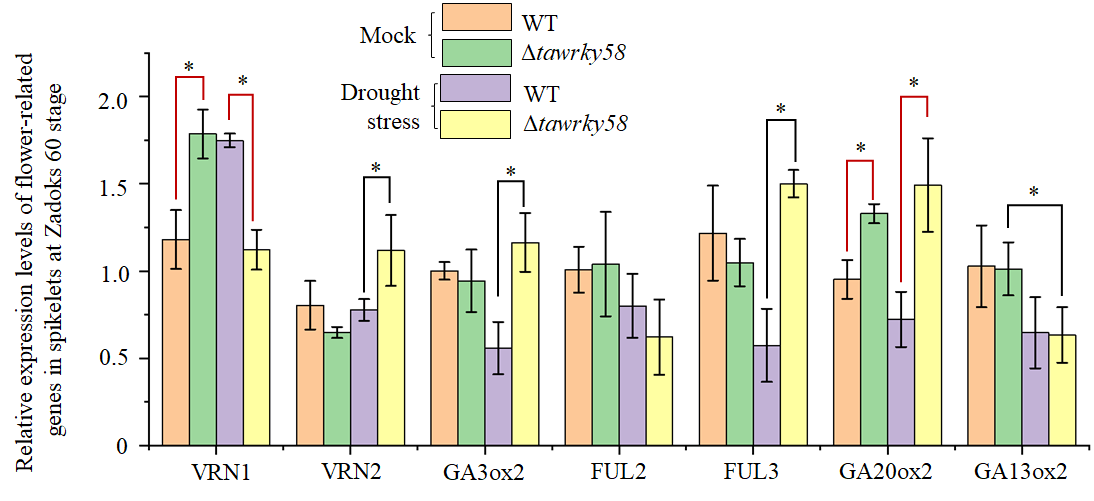

Supplement: S6 Fig — Asterisks (*) above bars indicate significant differences (P ≤ 0.05) relative to controls (Student’s t-test). Three biological replicates were performed for each plant. (TIF) [file pgen.1012124.s007.tif]

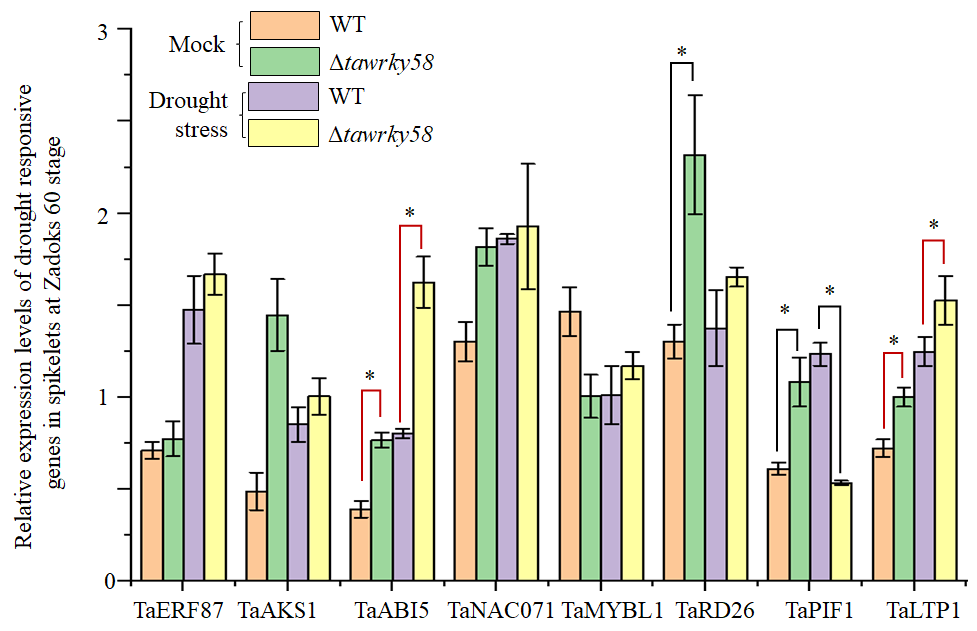

Supplement: S7 Fig — Asterisks (*) above bars indicate significant differences (P ≤ 0.05) relative to controls (Student’s t-test). Three biological replicates were performed for each plant. (TIF) [file pgen.1012124.s008.tif]

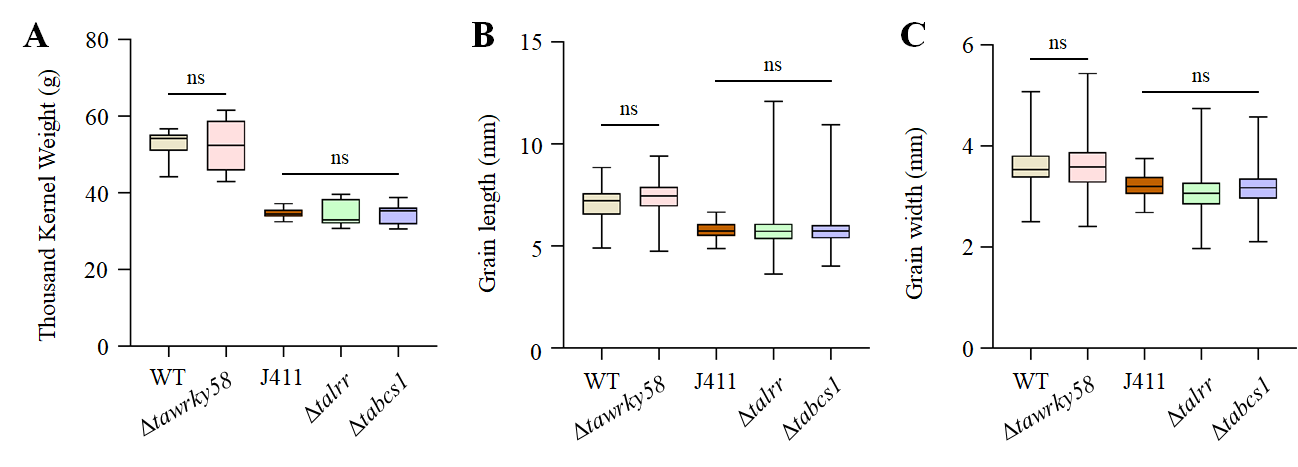

Supplement: S8 Fig — Thousand kernel weight (A), grain length (B), and grain width (C) were measured for the indicated genotypes. No significant differences were observed between any of the mutants and their respective wild-type controls (Student’s t-test, P > 0.05). Data are presented as mean ± SD (n ≥ 10). (TIF) [file pgen.1012124.s009.tif]

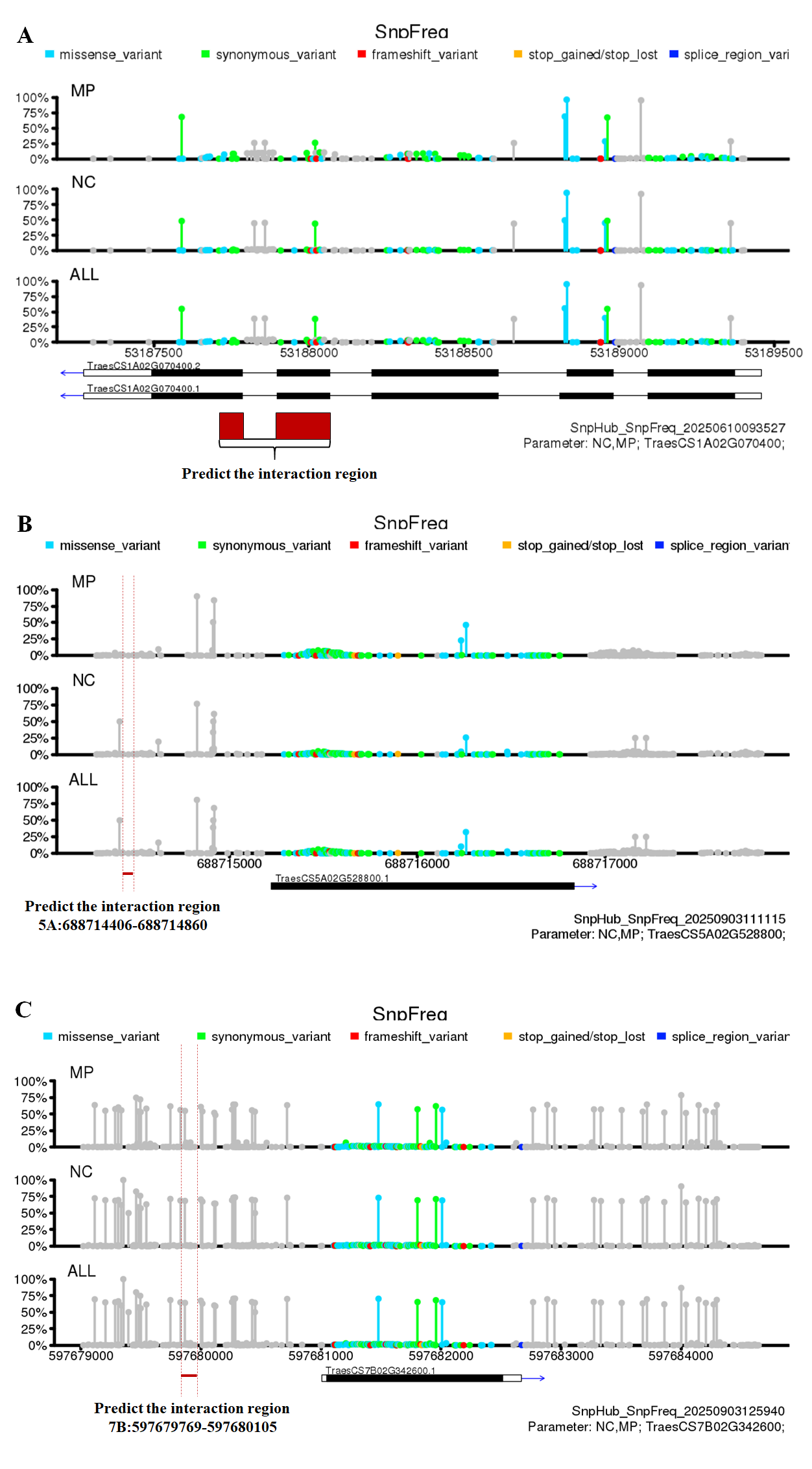

Supplement: S9 Fig — (TIF) [file pgen.1012124.s010.tif]
