# Supplementary material for: The transcription factor TaWRKY58 coordinates growth and drought sensitivity in wheat by repressing TaLRR and TaBCS1
Source: PLoS Genet. 2026 Apr 24;22(4):e1012124. doi: 10.1371/journal.pgen.1012124 (PMC13132433; doi:10.1371/journal.pgen.1012124)
Supplement: Attachment — Submitted filename: Author response to reviewer.pdf [file pgen.1012124.s015.pdf]

Reviewer #2: This manuscript presents a well-structured and significant study. The authors convincingly identify TaWRKY58 as a key transcriptional repressor that fine-tunes the trade-off between plant height and drought response in wheat. The combination of genetic, molecular, and phenotypic analyses is a major strength. The proposed regulatory module is novel and has clear implications for wheat breeding. However, few comments listed in below:

1. While the direct repression of TaLRR and TaBCS1 by TaWRKY58 is solidly demonstrated, the functional link between these targets and the observed phenotypes could be more deeply explored. The manuscript states TaLRR is a signaling protein and TaBCS1 is an energy metabolism protein, but how exactly their repression by TaWRKY58 leads to altered GA levels (for TaLRR?) and soluble sugar accumulation (for TaBCS1?) remains somewhat speculative. Thus, it will be good if co-authors could add data or a more detailed discussion on: for example, Does TaLRR mutation affect GA biosynthesis/signaling genes or pathways? Does TaBCS1 mutation directly impact mitochondrial function (e.g., ATP levels, ROS) or the expression of sugar metabolism genes under drought? Measuring GA levels in the Δtahr mutant and soluble sugars/energy status in the Δtabcs1 mutant would significantly strengthen the causal chain from TF to target to metabolic phenotype.

Author response:

We thank the reviewer for highlighting the need to strengthen the mechanistic link between the direct targets and the observed phenotypes. In response, we have now directly measured gibberellin (GA) levels in the Δ*talrr* and Δ*tabcs1* mutants under drought stress. Both mutants exhibited significantly reduced GA accumulation (new Fig. 7H), which strongly corroborates their dwarf and drought-sensitive phenotypes and provides direct evidence that these targets influence GA homeostasis. Furthermore, the reduced soluble sugar content in the Δ*tabcs1* mutant (Fig. 7G) supports a functional connection to energy metabolism.

We have expanded the Discussion (Page 17, lines 369–381) to propose distinct, testable pathways through which each target may affect GA levels. For TaLRR, we hypothesize

that it may modulate GA signaling or feedback regulation, given its leucine-rich repeat domain commonly involved in signal transduction. For *TaBCS1*, we suggest that its mitochondrial AAA$^+$ ATPase activity could influence cellular energy status, thereby impacting GA biosynthesis and sugar accumulation. We acknowledge that further experiments—such as transcriptomic analysis of GA biosynthesis genes in Δ*talrr* or mitochondrial functional assays in Δ*tabcs1*—are needed to fully elucidate these mechanisms, and we have indicated these as future research directions in the revised Discussion. We believe the new data and refined discussion significantly strengthen the causal chain from transcription factor to target to metabolic phenotype.

2. The observation that TaWRKY58 is stress-induced but its loss-of-function does not confer strong stress susceptibility (Fig. 4) is intriguing and well-discussed. However, the proposed "priming" or "buffering" hypotheses, while reasonable, are not directly tested. It will be good if the co-authors could analyze the kinetics of target gene repression (TaLRR, TaBCS1) following stress induction of TaWRKY58. Does transient TaWRKY58 induction lead to sustained repression of its targets? This would provide molecular evidence for a priming mechanism.

Author response:

We thank the reviewer for raising this important point. We agree that a detailed kinetic analysis of target gene expression following stress induction would provide valuable molecular insights into the proposed priming mechanism. While we are unable to perform these additional experiments at this stage due to resource constraints, we have thoroughly revised our manuscript to address this comment by reinterpreting our existing data and refining our discussion.

In the Discussion section (Page 17, lines 340–368), we have significantly reorganized and expanded our discussion of this phenomenon. We now present the “priming” and “buffering” hypotheses as the most logical interpretations of our current dataset, while explicitly acknowledging that they remain to be tested dynamically. We have added the following sentences to clarify our model:

"The expression patterns of its target genes under stress further support this model. While *TaWRKY58* is induced, its targets *TaLRR* and *TaBCS1* are ultimately upregulated under prolonged drought in wild-type plants (Fig. 7E). This suggests that the initial, transient repression by *TaWRKY58* is eventually overridden by stronger, direct stress-responsive transcriptional activators—a classic feature of a regulatory module that integrates multiple signals to fine-tune outputs."

We conclude this section by emphasizing that our steady-state mutant analyses firmly establish *TaWRKY58* as a key repressor within this integrated network, and we identify time-course expression profiling under controlled stress conditions as a critical future direction to validate the proposed regulatory dynamics. We believe these revisions directly address the reviewer's concern by providing a more nuanced and data-driven interpretation without necessitating additional experiments.

3. The drought tolerance data for the Δtawrky58 mutant is based on soluble sugar accumulation and survival cell counts (S5 Fig). More comprehensive physiological data would bolster the claim of "enhanced drought tolerance." It will be good if co-authors could Include standard drought physiology metrics for the key genotypes (WT, Δtawrky58, overexpression line), such as: Water loss rate (relative water content over time), Stomatal conductance or photosynthetic parameters under progressive drought, or a clearer yield component assessment (e.g., spike fertility, grain number/weight) after drought-recovery cycles, especially given the focus on "yield stability."

Author response:

We thank the reviewer for this valuable suggestion. We agree that comprehensive physiological metrics would further substantiate the drought tolerance phenotype. In response, we have taken several steps to strengthen our argument within the constraints of the current study.

First, we have revised the Discussion (Page 16, lines 332–339) to explicitly link our experimental data—including soluble sugar accumulation, cell viability, and GA measurements—to established drought physiology indicators such as osmotic

adjustment capacity and tissue integrity. Soluble sugar accumulation is widely recognized as a reliable indicator of osmotic adjustment, which directly contributes to water retention and membrane stability under drought stress (Kaur et al., 2021; Hou et al., 2018). The increased cell viability observed in the Δ*tawrky58* mutant (S5 Fig) further supports reduced cellular damage, analogous to electrolyte leakage assays commonly used in drought studies. Together with our phenotypic observations and GA measurements under drought, these data collectively support enhanced drought tolerance in the mutant.

Second, we fully acknowledge that field-based yield assessment under drought is essential for breeding applications. While multi-location and multi-year trials are beyond the scope of the current study due to restrictions on large-scale planting of transgenic materials in China, we have evaluated key yield-related traits—including thousand kernel weight, grain length, and grain width—in the Δ*tawrky58*, Δ*talrr*, and Δ*tabcs1* mutants. Notably, none of these mutations significantly affected the measured yield traits (S8 Fig), suggesting that manipulation of this regulatory module may offer a promising strategy for improving drought tolerance without compromising yield potential.

These revisions clarify the physiological basis of drought tolerance in our system and highlight the potential of *TaWRKY58*, *TaLRR*, and *TaBCS1* as valuable targets for wheat breeding programs aimed at enhancing stress resilience.

Reference:

Kaur H, Manna M, Thakur T, Gautam V, Salvi P. Imperative role of sugar signaling and transport during drought stress responses in plants. Physiol Plant. 2021 Apr;171(4):833-848. doi: 10.1111/ppl.13364. Epub 2021 Mar 1. PMID: 33583052.

Hou J, Huang X, Sun W, Du C, Wang C, Xie Y, Ma Y, Ma D. Accumulation of water-soluble carbohydrates and gene expression in wheat stems correlates with drought resistance. J Plant Physiol. 2018 Dec;231:182-191. doi: 10.1016/j.jplph.2018.09.017. Epub 2018 Sep 27. PMID: 30278314.

4. The figure legends often state "n ≥ 3 biological replicates" or similar. For critical phenotypic measurements (e.g., plant height, soluble sugar content), the

exact 'n' for each genotype should be explicitly stated in the legend or figure. The "Material and Methods" states two independent experimental replicates were conducted; this should be clarified in the context of biological vs. technical replication.

Author response: We apologize for the lack of clarity. We have revised all figure legends throughout the manuscript to now explicitly state the exact number of biological replicates (n) used for each experiment. For instance, the legend for Fig. 3D now reads: "Plant height measurements at Zadoks stage 65 for wild-type (WT) and Δtawrky58 homozygous lines (n = 15 plants per genotype)." We have also removed the redundant phrase regarding "two independent experimental replicates" from the Materials and Methods section to avoid confusion, as suggested.

5. Fig 3H & 4J: Please specify which specific GA(s) were measured (e.g., GA1, GA4).

Fig 4: The legend for panels H, I, J references "figure 4H." This is confusing. Please correct the labels (likely should be "in plants from H" or similar).

Author response:

We have addressed these points as follows:

GA Specification: We have clarified in the Materials and Methods section that the ELISA kit used in our study specifically detects GA1, a key bioactive GA involved in stress responses. The figure legends for Fig. 3H and Fig. 4J have been updated to "GA1 content."

Fig. 4 Legend: We have corrected the confusing labels. The legend for panel J now reads: "J. GA1 content in spikelets and rachis of plants from panel H."

Fig. 6A: We appreciate the reviewer's suggestion. However, given that the overexpression line (O-*TaWRKY58*-16) phenocopies the WT in terms of plant height, GA levels, and drought response (as shown in Fig. 3 and Fig. 4), and serves to complement the Δ*tawrky58* mutant phenotype, we believe that including its expression data for *TaLRR* and *TaBCS1* in Fig. 6A would be redundant and would not alter the conclusions. We respectfully prefer to keep the figure focused on the mutant data to maintain clarity. The repressive role of *TaWRKY58* is firmly established by the EMSA,

dual-LUC assays, and the increased expression of these targets in the mutant (Fig. 6A, D, E, F, G).

6. Page 13, Line 200-201: "We detected 754,059 TaWRKY58-bound peaks" – This number seems extremely high. Please clarify if these are raw peaks from sequencing or after stringent filtering. A brief comment on what this high number might imply (e.g., low binding specificity, or a truly global regulator) would be useful.

Author response:

We thank the reviewer for the opportunity to clarify this point. The 754,059 peaks represent the high-confidence peaks identified after stringent filtering ($Q < 0.05$). We have now added a clarifying sentence in the Results section (page 10, lines 204–205) to state this explicitly.

We also appreciate the reviewer's request for a brief interpretation of this high number. Several factors may contribute to the large number of peaks: (i) the wheat genome is exceptionally large (~16 Gb), and the DNA input in DAP-seq experiments is typically scaled accordingly, which can increase the number of detectable binding events; (ii) *TaWRKY58* appears to function as a global transcriptional regulator, and its strong DNA-binding affinity likely results in the pulldown of numerous specific fragments; and (iii) the high-quality data obtained in our experiment, combined with a relatively lenient peak-calling threshold after stringent filtering, may also contribute to the observed peak count. Collectively, these observations suggest that *TaWRKY58* indeed binds to a large set of target sites across the wheat genome, consistent with its role as a pleiotropic regulator.

7. Page 16, Line 283-284: "Under drought stress, the expression levels of TaLRR and TaBCS1 were significantly upregulated..." This seems counterintuitive if TaWRKY58 is induced and represses them. Please clarify or discuss this point—it might indicate a feedback loop or stress-induced override of repression.

Author response: This point is related to Comment 2. As detailed in our response to Comment 2, we have addressed this apparent paradox by revising the Discussion sections. We now explicitly discuss that the upregulation of *TaLRR* and *TaBCS1* under

prolonged drought in wild-type plants (Fig. 7E) indicates that their expression is under complex control. While *TaWRKY58* can repress them, other stronger, stress-activated transcriptional activators likely dominate under sustained stress conditions. This regulatory logic positions *TaWRKY58* as a modulator of basal expression levels, which fine-tunes the plant's preparatory state, rather than as the primary driver of the acute stress response. This interpretation is now clearly integrated into our model. And We also illustrated Figure 7E as “E. the expression levels of *TaLRR* and *TaBCS1* in J411 leaves after 7 days of 20% PEG6000 treatment at Zadoks stage 13.”

6. The discussion is strong and thoughtful. The model (Fig. 8) is helpful but could be slightly enhanced. Consider adding the metabolic outputs (GA, soluble sugars) and the resulting phenotypes (height, drought tolerance) to the schematic to make the pathway from molecular mechanism to agronomic trait even clearer.

Author response: We appreciate the positive feedback and the helpful suggestion. We have revised Fig. 8 to make the pathway from molecular mechanism to agronomic trait clearer. The updated schematic now explicitly includes the metabolic outputs ("GA accumulation" and "Soluble sugar accumulation") and connects them to the resulting phenotypes ("Increased plant height / Early flowering" and "Drought tolerance"). We have used distinct colors to differentiate the signaling pathway (involving *TaLRR*) and the energy metabolism pathway (involving *TaBCS1*). The figure legend has been updated accordingly.

7. DAP-seq: The method states genomic DNA was from 'Fielder,' but the study uses 'Kronos' and 'Jing411' mutants. Please justify the use of a different cultivar for this genome-wide assay and discuss potential limitations.

Author response:

We thank the reviewer for this important observation. We used 'Fielder' (hexaploid, AABBDD) for DAP-seq because: (1) it contains all three subgenomes, including the D genome present in our hexaploid mutant 'Jing411' (AABBDD); (2) the core DNA-binding specificity of WRKY transcription factors is highly conserved across wheat cultivars, with W-box motifs showing minimal variation; and (3) 'Fielder' is a widely used cultivar for functional genomic studies in wheat. Its use allows DAP-seq to capture

TaWRKY58 binding sites across all three subgenomes (A, B, and D), providing a more comprehensive view of its regulatory targets in hexaploid wheat.

Importantly, all key interactions were experimentally validated using EMSA and dual-luciferase assays (Fig 6D–G), confirming that the identified binding events are biologically relevant to the genetic backgrounds used for phenotypic analysis.

We have added a brief statement in the Materials and Methods (Page 20, lines 450-455) to clarify the rationale and acknowledge this as a minor limitation.

“DAP-seq was performed using genomic DNA from the hexaploid wheat cultivar 'Fielder' (AABBDD) to capture binding sites across all three subgenomes, including the D genome present in the hexaploid mutant 'Jing411'. Given that WRKY DNA-binding domains recognize highly conserved W-box motifs across wheat cultivars, this approach allows genome-wide identification of *TaWRKY58* binding sites while maintaining alignment compatibility with the 'Chinese Spring' reference genome.”

We appreciate for Reviewers’ warm work earnestly, and hope the correction will meet with approval. Once again, thank you very much for your comments and suggestions.

Sincerely,

Yazhou Zhang
